# The Influence of Orthographic Neighborhood Density and Word Frequency on Visual Word Recognition: Insights from RT Distributional Analyses

**DOI:** 10.3389/fpsyg.2016.00401

**Published:** 2016-03-31

**Authors:** Stephen Wee Hun Lim

**Affiliations:** Department of Psychology, Faculty of Arts and Social Sciences, National University of SingaporeSingapore, Singapore

**Keywords:** distributional analyses, orthographic neighborhood density, visual lexical decision, visual word recognition, word frequency

## Abstract

The effects of orthographic neighborhood density and word frequency in visual word recognition were investigated using distributional analyses of response latencies in visual lexical decision. Main effects of density and frequency were observed in mean latencies. Distributional analyses additionally revealed a density × frequency interaction: for low-frequency words, density effects were mediated predominantly by distributional shifting whereas for high-frequency words, density effects were absent except at the slower RTs, implicating distributional skewing. The present findings suggest that density effects in low-frequency words reflect processes involved in early lexical access, while the effects observed in high-frequency words reflect late postlexical checking processes.

## Introduction

Word frequency and orthographic neighborhood density effects are among the most influential findings in the visual word recognition literature. In models of visual word recognition, the goal of processing is often referred to as lexical access or lexical retrieval (see Davis, 2010, for a comprehensive review). These models postulate mechanisms and representations involved in the processing of visual orthographic stimuli (Protopapas and Kapnoula, 2015), which are typically studied using naming and lexical decision tasks (LDTs) that require lexicality discrimination and decision where subjects would classify stimuli as either words or non-words, and the speeded pronunciation (word naming) task that involves lexical access but excludes the word/non-word discrimination and decision components of the LDT. Researchers have investigated the effects of both lexical variables and such sublexcial ones as neighborhood density and word frequency on response time (RT) distributions (see reviews by Balota et al., 2006, 2012). They have further conducted multivariate analyses of several of these variables studied concurrently (Yap and Balota, 2009) and on substantive databases (e.g., Balota et al., 2007; Ferrand et al., 2010; Keuleers et al., 2010, 2012). The resultant effects have, in turn, been useful for assessing visual word recognition models as well as pursuing theoretically interesting questions (e.g., Coltheart et al., 2001; Reynolds and Besner, 2002, 2004; Mulatti et al., 2006; Perry et al., 2007, 2010; Adelman and Brown, 2008a,b).

Word frequency effects, where latencies for common words are faster than those that are relatively less common, have been observed in many LDT studies (see Balota and Chumbley, 1990, for a classic review). In visual word recognition, frequency effects have been attributed to changes in activation thresholds or baselines. The logogen-style activation framework was inaugurated by Morton (1969), which assumes that information extracted from the sensory representation of the word leads to parallel activation of all word units that match that information. When sufficient activation has accumulated in a particular word unit, it reaches threshold and lexical access occurs. Morton's (1969) initial model was later specified in greater detail by McClell and Rumelhart (1981). Their model, which they called the interactive activation model, suggests that activation occurs at three levels. Activation of featural units feeds to units corresponding to letters, which in turn activate the units for words containing these letters. Activity also feeds back from the word to the letter level, causing reverberating patterns of activity to occur between these levels. To ensure that only one word unit eventually obtains threshold, McClell and Rumelhart (1981) also assume that inhibition occurs between word units, so that the activity level of competing word units is reduced relative to the maximally active node. Within the activation framework, word frequency is assumed to be reflected in the threshold (Morton, 1969) or resting activation level (McClell and Rumelhart, 1981) associated with a particular word unit. The critical interpretation is that less evidence is required to enable recognition of a high-, than a low-, frequency word.

The findings for orthographic neighborhood density effects (N), on the other hand, appear to be more mixed. The N metric has been defined by Coltheart et al. (1977) as the number of close neighbors a word has and refers to the number of words that can be created by changing a single letter of this target word. For instance, *tell* has many neighbors such as *well, yell, sell, teal*, and *tall*, while *once* has no neighbors. Neighborhood effects can help specify the mechanisms underlying lexical access. The implication of the overlap in the features constituting different words is that any subset of the features constituting a particular word is unlikely to uniquely specify its corresponding lexical representation. Neighbors are items that are highly confusable with the target word, in the sense that they share a large number of their features with the target. Thus, it seems inevitable that some or all of the neighbors of a target word will be selected by the access mechanisms as eligible target candidates.

Effects of N can be accommodated within activation-based models of lexical access, and appear to provide substantive support for an activation mechanism. If presenting a word leads to an activation of all lexical items that sufficiently match features of the target word, the density of the word's neighborhood should influence access time. Unfortunately, this class of models does not make precise predictions about the nature of the effect of neighborhood density. McClell and Rumelhart's (1981) interactive activation model, for instance, assumes excitatory links between levels which can account for facilitatory effects of neighborhood size. Activated neighbors will feed back to their constituent letters which in turn lead to heightened activation of word units containing these letters. According to McClell and Rumelhart (1981), such facilitatory effects of N are likely to be greater for low- than high-frequency words. The reason is that high-frequency words have higher base activation levels and are therefore likely to reach threshold before allowing reverberating letter-level activation from neighboring word units to become influential.

Yet, the same model can also predict inhibitory effects of neighborhood size because of its assumption of lateral inhibition between word nodes. Active nodes send inhibition to other active nodes to an extent that is proportional to their current activation. If the unit corresponding to the target word becomes activated before other units, this inhibitory mechanism would decrease background activation and make the target more salient. On the other hand, if nodes corresponding to neighbors obtained activation before the target word, these activated competitors would inhibit activation of the target and delay threshold activation. The more neighbors a word has, the greater the likelihood that the target unit would fall prey to this inhibitory mechanism, resulting in interfering effects of large neighborhoods. Thus, depending on the relative contribution to performance of excitatory activation between letter and word levels, as well as inhibitory activation within the lexical level, the interactive activation model can explain facilitatory, inhibitory, or null effects of neighborhood size.

Using the visual LDT paradigm, Coltheart et al. (1977) first observed that low-N non-words were classified more quickly than high-N non-words, but that N did not influence performance for English words. The researchers interpreted their data using Morton's (1969) logogen-style activation framework, in which the strength of activation in individual logogens is determined by sensory input and is insensitive to activity in other logogens. The researchers then attributed N effects on non-word classification to a decision mechanism that is sensitive to the overall lexical activation. Subsequently, Andrews (1989) reported that N actually influenced responses to English words in the LDT when the words were selected to orthogonally manipulate N and word frequency. Specifically, it was reported that high N facilitated performance for words, but only for the 4-letter low-frequency words. These facilitatory effects of N, which are not incompatible with McClell and Rumelhart's (1981) interactive activation model, were later replicated in several other experiments (e.g., Andrews, 1992; Michie et al., 1994; Sears et al., 1995). However, Grainger et al. (1989) concurrently found no systematic relationship to exist between N and performance in the LDT; lexical decision latencies were not affected by the number of neighbors *per se*.

Visual lexical decision studies that examined neighborhood effects have traditionally used mean RT differences among the experimental conditions to make inferences about the mechanisms underlying the recognition process. The implicit assumption that the researchers would have made is that RT distributions across conditions are symmetrical, where the mean constitutes a reasonably good estimate of the central tendency of these distributions. But RT distributions are in fact rarely symmetrical around a mean. They typically assume a positively skewed unimodal shape which contains information that cannot be derived from the mean and variance of the distributions. For instance, mean RT differences, or the lack thereof, between conditions can be due to changes in the shape (skew) of the distribution in itself or in addition to a shift in the modal portion of the distribution. By relying on a traditional RT analysis that uses mean RTs as the dependent variable (DV) to interpret LDT performance, one can, in some instances, fail to recognize the tradeoff between the effects of shifting and skewing, and be misled to incorrectly infer null results (Heathcote et al., 1991). Recognizing the problems concerned with the traditional RT analysis approach, several researchers have argued that the nature of the RT distributions ought to be scrutinized more closely (e.g., Heathcote et al., 1991; Balota et al., 2008).

Two distributional analyses techniques were used in the present study, namely the ex-Gaussian and Vincentile analyses. Shifting and skewing in the RT distributions were investigated using the ex-Gaussian function. The procedure was to fit an empirical RT distribution to this theoretical function that captures important aspects of typical RT distributions. The ex-Gaussian function conceptualizes RT distributions as the convolution of two underlying distributions: a Gaussian distribution and an exponential distribution. This yields three parameter estimates: mu (mean of the normal distribution), sigma (standard deviation of the normal distribution), and tau (mean and standard deviation of the exponential component). An important property of the ex-Gaussian function is that the mean of the RT distribution is constrained to be the algebraic sum of the mu and tau parameters obtained by fitting that distribution. This constraint allows one to partition mean differences into individual components due to distributional shifting (mu) and skewing (tau), and then make inferences from these components to determine the nature of the effect of an independent variable (IV) (see Balota et al., 2008; Yap and Seow, 2014).

Parameter estimates from the ex-Gaussian function were supplemented by analyses of Vincentiles to enable a graphical, non-parametric estimate of the variable's effect. In these analyses, the RTs are ordered, from fastest to slowest, within each condition, and the average of the first 10%, that of the second 10%, and so forth, are plotted. The mean of the Vincentiles across participants can then be plotted to obtain a description of how the RT distribution is changing across conditions. Importantly, differences between two levels of an IV across Vincentiles can be graphically represented to reveal how the effect of an IV may change across different portions in the RT distribution.

In this study, we pursued two goals. The first was to replicate the N effects in the visual LDT in the light of the initial contradictory reports (see Coltheart et al., 1977; Andrews, 1989, 1992; Grainger et al., 1989). The present hypothesis was that facilitatory effects of density would be observed, but only for low-frequency words (cf. Michie et al., 1994; Andrews, 1989, 1992; Sears et al., 1995). The second, and more important, goal was to extend the ex-Gaussian and Vincentile analyses techniques to the orthographic neighborhood density and word frequency effects found in the extant visual lexical decision studies, and to explore the extent to which these two effects are driven by distributional shifting and skewing.

## Method

### Participants

Fifty-seven introductory psychology students from the National University of Singapore with no reported history of speech or hearing impairment participated for course credit. Their mean vocabulary age of the Shipley Test was 18.09 (*SD* = 1.06). This research was conducted with the appropriate ethics review board approval by the National University of Singapore, and participants have granted their written informed consent.

### Design and materials

A 2 (Neighborhood Density: low, high) × 2 (Word Frequency: low, high) within-subjects design was used. Forty 4-letter English words were selected for each of the four conditions, and their properties are summarized in Table 1. The range of orthographic neighbors (ONs) for low-density words was 0 to 6, whereas the range of ON for high-density words was 11 to 20. Two-way analyses of variances (ANOVAs) showed a main effect of frequency, *F*_(1, 156)_ = 19826.68, *MSe* = 0.67, *p* < 0.001, for the log-frequency values (*M* = 6.58, *SD* = 0.53 for low-frequency words and *M* = 11.67, *SD* = 1.02 for high-frequency words), and a main effect of density, *F*_(1, 156)_ = 1827.88, *MSe* = 2.10, *p* < 0.001 for the density values (*M* = 3.35, *SD* = 1.38 for low-density words and *M* = 13.14, *SD* = 1.50 for high-density words). No other effects were significant, *Fs* < 1. The 160 legal non-words used were obtained from the ARC non-word database (Rastle et al., 2002) and were matched against the 160 words in terms of length and density (see Supplementary Material).

**Table 1:** **Mean density and log-frequency of the words in the neighborhood density and word frequency conditions**.

**Conditions**	**Density**		**Log-frequency**	
	***M***	***SD***	***M***	***SD***
**LOW-FREQUENCY**				
Low-density	3.33	1.33	6.61	0.54
High-density	13.05	1.95	6.56	0.52
**HIGH-FREQUENCY**				
Low-density	3.38	1.44	11.67	1.23
High-density	13.23	0.86	11.67	0.78

### Procedure

Participants were tested on individual PCs in groups of seven or fewer. E-prime 1.2 and the PST Serial Response Box (Schneider et al., 2002) were used for stimuli presentation and data collection. Participants were instructed to indicate as quickly and as accurately as possible whether the visual token presented on each trial was a real English word (or a non-word). The left- and right-most buttons of the button-box were labeled *No* and *Yes*, respectively. On each trial, a fixation cross appeared and remained on the screen for 500 ms, and terminated for 200 ms before the target word appeared. RT was measured from the onset of the target stimulus to the button-press. Accuracy feedback was provided for each trial. A practice set of 20 trials for task familiarization was given, using stimuli unrelated to the experiment. The 320 experimental trials were then presented in a random order for each participant, with a short self-paced break after every set of 80 trials was completed.

## Results

Errors and latencies faster than 200 ms or slower than 3000 ms were first excluded, and the overall word and non-word means and SDs for each participant were computed across all conditions. Following which, latencies exceeding 2.5 SDs from the participant mean, as well as items where proportion of correct responses was not at least 0.49 (DILL, GAGE, and HICK), were removed. Table 2 summarizes the results obtained from mean latencies, accuracy, and the ex-Gaussian parameters. Two way ANOVAs by participants (*F*_*p*_) and items (*F*_*i*_) were performed for latencies and accuracy, and by participants for the ex-Gaussian parameters.

**Table 2:** **Mean latency, accuracy, and ex-gaussian parameter estimates across neighborhood density and word frequency**.

**Conditions**	**Latency**	**Accuracy**	**Mu**	**Sigma**	**Tau**
**LOW-FREQUENCY**					
Low-density	679 (123)	87 (11)	535 (79)	59 (38)	147 (89)
High-density	662 (127)	88 (8)	509 (74)	54 (37)	157 (84)
Density effect	17	−1	26	5	−10
**HIGH-FREQUENCY**					
Low-density	554 (90)	98 (2)	444 (45)	35 (14)	112 (62)
High-density	546 (83)	99 (1)	442 (47)	38 (16)	105 (54)
Density effect	8	−1	2	−3	7
Interaction	9	0	24	8	−17
Non-words	692 (144)	94 (4)	542 (68)	58 (23)	152 (90)

*SDs in parentheses*.

### Latency

For latency, reliable main effects of density, *F*_*p*__(1, 54)_ = 11.51, *MSe* = 790.68, *p* < 0.01, and frequency, *F*_*p*__(1, 54)_ = 222.87, *MSe* = 3600.78, *p* < 0.001, were obtained for the analyses by participants. Participants were faster in responding to high-density words (*M* = 604, *SD* = 102) than to low-density words (*M* = 617, *SD* = 104); they were also faster in responding to high-frequency words (*M* = 550, *SD* = 83) than to low-frequency words (*M* = 671, *SD* = 123). For the analyses by items, a reliable main effect of frequency was obtained, *F*_*i*__(1, 153)_ = 299.53, *MSe* = 1981.84, *p* < 0.001. High-frequency words yielded a shorter response time (*M* = 551, *SD* = 30) as compared to low-frequency words (*M* = 674, *SD* = 56). No other effects were significant, *Fs* < 2.01, *MSes* < 1982.84, *ps* > 0.1.

### Accuracy

For accuracy, there was a reliable main effect of frequency, *F*_*p*__(1, 54)_ = 90.97, *MSe* = 0.007, *p* < 0.001 for the analyses by participants; the main effect of density was marginally significant, *F*_*p*__(1, 54)_ = 3.53, *MSe* = 0.001, *p* = 0.066. Participants were more accurate with high-frequency words (*M* = 98, *SD* = 0.01) than with low-frequency words (*M* = 88, *SD* = 0.09); they also tended to be more accurate with high-density words (*M* = 93, *SD* = 0.04) than with low-density words (*M* = 92, *SD* = 0.06). For the analyses by items, a reliable main effect of frequency was obtained, *F*_*i*__(1, 153)_ = 55.86, *MSe* = 0.008, *p* < 0.001. High-frequency words yielded a higher accuracy rate (*M* = 98, *SD* = 4) as compared to low-frequency words (*M* = 88, *SD* = 12). No other effects were significant, *Fs* < 1.16, *MSes* < 0.008, *ps* > 0.1.

### Mu

Turning to the ex-Gaussian parameters, for mu, there were reliable main effects of density, *F*_(1, 54)_ = 18.61, *MSe* = 589.63, *p* < 0.001, and frequency, *F*_(1, 53)_ = 160.02, *MSe* = 2151.95, *p* < 0.001. These main effects were qualified by the significant interaction, *F*_(1, 53)_ = 12.00, *MSe* = 726.81, *p* < 0.01. Simple main effects analyses at each level of the frequency factor revealed that for low-frequency words, mu was larger for low-density words compared to high-density words, *F*_(1, 54)_ = 16.56, *MSe* = 1185.71, *p* < 0.001, but there was no density difference for high-frequency words, *F* < 1. This finding implicates a shift in the modal portion of the RT distribution as a function of density, but only for low-frequency words.

### Sigma

For sigma, a significant main effect of frequency was obtained, *F*_(1, 54)_ = 25.75, *MSe* = 890.57, *p* < 0.001. Sigma was larger for low-frequency (*M* = 57, *SD* = 29) than high-frequency (*M* = 36, *SD* = 13) words. No other effects were significant, *Fs* < 1.32, 0*MSes* < 645.75, *ps* > 0.1.

### Tau

For tau, a significant main effect was obtained for frequency, *F*_(1, 54)_ = 37.25, *MSe* = 2834.02, *p* < 0.001, but not for density, *F* < 1. The main effect of frequency appears to be qualified by the marginally significant interaction, *F*_(1, 54)_ = 3.21, *MSe* = 1417.98, *p* = 0.079. Follow-up analyses indicated that tau tends to be smaller for low-density words compared to high-density words for low-frequency words, but it tends to be larger for low-density words compared to high-density words for high-frequency words. More important, a cross examination of the tau data, with the mu data, revealed that the small density effect observed for the high-frequency word condition appears to be attributable to distributional skewing, rather than distributional shifting.

Recall that one important constraint of the ex-Gaussian analyses is that the mean of the RT distribution is the algebraic sum of mu and tau. In the traditional mean latency analyses, only reliable main effects of frequency and density were obtained; there was no reliable frequency × density interaction. Analyses of the ex-Gaussian parameters provide important observations that constitute a more faithful account of the apparent lack of interaction between the factors.^1^ First, analyses of the mu parameter as a function of density suggest that there is distributional shifting only for the low-frequency words but not for the high-frequency words. This finding strongly suggests that the density effect observed for low-frequency words in the traditional mean latency analyses is predominantly mediated by distributional shifting. Second, analyses of the tau parameter, in conjunction with the mu parameter, strongly suggest that the small density effect observed for high-frequency words in the traditional mean latency analyses is, on the other hand, largely mediated by distributional skewing.

**Figure 1:**
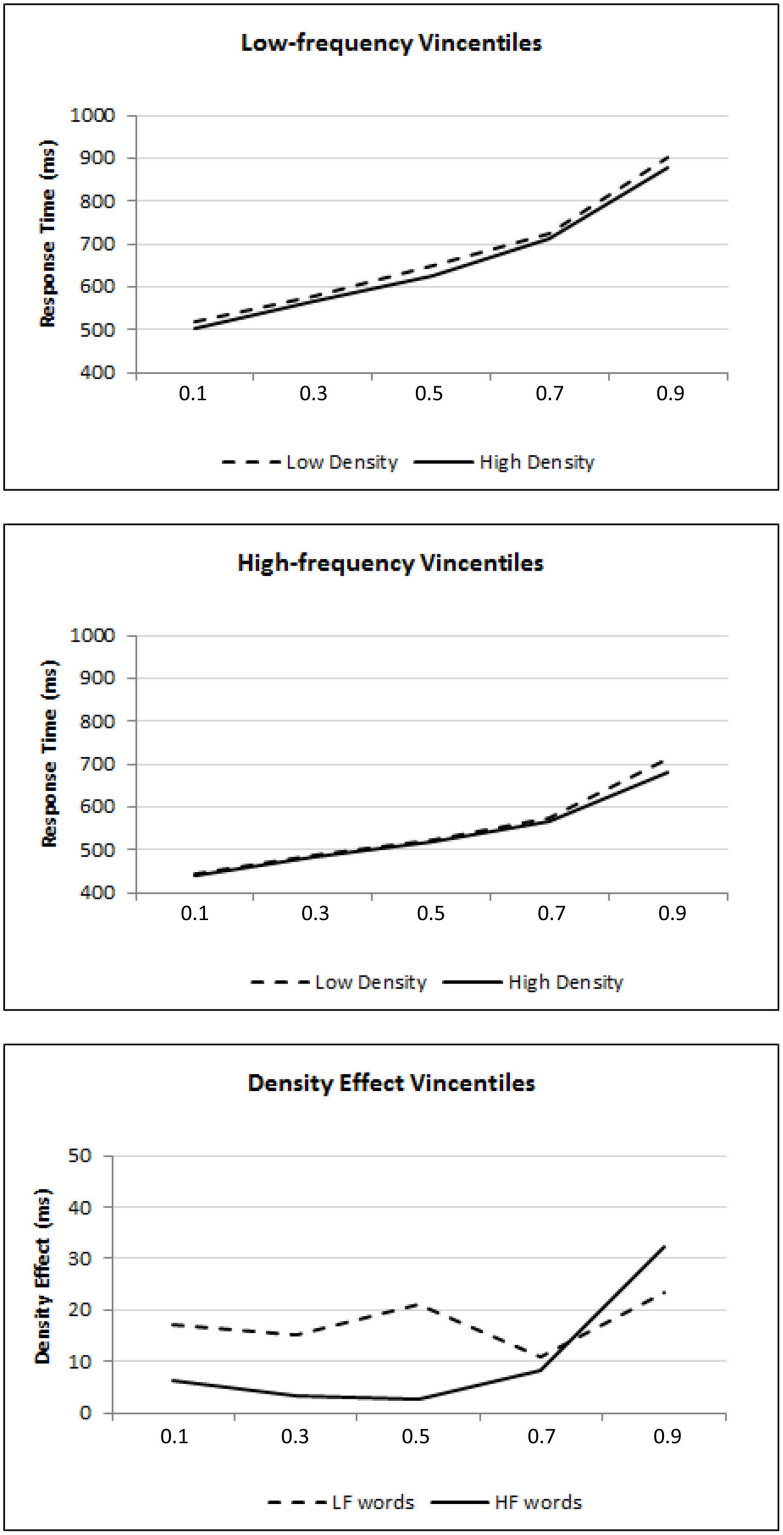
**Vincentiles of lexical decision performance**. The participants' mean vincentiles are represented across different conditions. The lines represent the estimated vincentiles of the best-fitting ex-Gaussian distribution. The top and middle panels show performance as a function of density in the low- and high-frequency conditions respectively, while the bottom panel shows the density effect.

To corroborate this interpretation, vincentile analyses were performed on the RT data. Figure 1 shows the mean vincentiles across the different experimental conditions.^2^ The lines represent the estimated vincentiles of the best-fitting ex-Gaussian distribution. This graphical representation allows a visual assessment of the goodness-of-fit between the empirical and estimated vincentiles.

From the top panel, it is clear that the density effect is observed for the low-frequency words across all vincentiles. The high-density means are always below the low-density means in each of the vincentiles. In the middle panel, the density effect is only apparent at the later vincentiles. The differential density effects can be seen more clearly in the bottom panel, which plots the difference scores between the low- and high-density means for each of the low- and high-frequency conditions. It can be observed that the density effect generally remains stable across vincentiles for the low-frequency words, indicating that the difference between low- and high-density words remains fairly constant as RT increases. This trend implicates distributional shifting *per se*. However, for high-frequency words, the density effect increases only in the slower RTs. This trend implies distributional skewing *per se*.

## Discussion

RT distributional analyses of orthographic neighborhood density and word frequency effects in visual lexical decision have not been done in previous studies examining neighborhood effects, which relied on *mean* RTs as the primary DV. The findings in the present study can be summarized as follows.

First, facilitatory effects of frequency, where high-frequency words elicited faster RTs than low-frequency words did, and of density, where words from high-density neighborhoods elicited faster RTs than words from low-density neighborhoods did, were obtained.

Second, and more important, the distributional analyses revealed a density x frequency interaction which was primarily attributable to differential shifting and skewing of the latency distribution between low- and high-density words as a function of frequency. For low-frequency words, the density effect obtained, replicating Andrews' (1989, 1992) finding, and the effect was predominantly mediated by distributional shifting; for high-frequency words, the small density effect observed was primarily mediated by distributional skewing.

A shift in the RT distribution as a function of density for low-frequency words is compatible with existing accounts which assume that lexical access relies upon an activation mechanism. Such an activation mechanism, which postulates top-down feedback from word to letter nodes, characterizes McClell and Rumelhart's (1981) interactive activation model^3^ which assumes parallel activation of both lexical units and units that correspond to sublexical components, such as letters. First, the assumption must hold that excitatory activation between lexical and sublexical units is not canceled out by lateral inhibition at the lexical level. Then, the partial activation of neighbors can increase the activation of sublexical components of the target, and consequently accelerate access to the target representation.

To explain the present data within such an activation mechanism framework, one must specify why the neighborhood effects arising from such sublexical/lexical interactions would affect only responses to low-frequency words. Frequency effects have mainly been attributed to differences in the resting activation level of lexical units within the original logogen (Morton, 1969) as well as the interactive activation (McClell and Rumelhart, 1981) accounts. A functionally equivalent assumption appears to characterize distributed memory models (McClelland and Rumelhart, 1985) that assume that frequency determines how rapidly a lexical unit reaches a threshold level of activation. The present interaction between frequency and neighborhood size implicates that sublexical units play a greater role in the recognition of low-, rather than high-, frequency words; high-frequency words obtain threshold sufficiently quickly through direct activation of lexical units, such that they are not influenced by the reverberating sublexical activation arising from active neighbors.

The increase in response time as a function of density for low-frequency words observed in the present study appears to be additive in nature, reflected by the distributional shift. Such a shift effect has been argued by Balota and Spieler (1999) to indicate early automatic processes, rather than later analytical or more attention-demanding processing. That density effects for low-frequency words are predominantly mediated by distributional shifting reflect processes involved in early lexical access, and not late postlexical processes which may also be involved in the LDT.

On the other hand, for high-frequency words, it appears that density effects are absent except at the slower end of the distribution, which are reflected in slightly greater skewing for low-density words. Recall that under the activation framework (e.g., McClelland and Rumelhart, 1985), high-frequency words obtain threshold sufficiently quickly through direct activation of lexical units, such that lexical *access* need not be facilitated by the reverberating sublexical activation arising from activated neighbors. The tau parameter revealed, for high-frequency words, some difference in RTs comparing low- with high-N words. It appears that high-frequency words with small neighborhoods would have received little facilitation from their active neighbors to aid lexicality *decision* of the target, as compared to those with big neighborhoods. Where facilitatory effects of N were lacking, compensatory postlexical checks could tend to be adopted, resulting in slightly longer RTs for low-N words. The emergence of density effects at the tail end of the distribution may therefore reflect, particularly for the low-N words, late postlexical checking processes that are specific to the lexical decision task (Balota and Chumbley, 1984), rather than early lexical access processes.

## Conclusion

The present study underscores the contribution of distributional analyses in illuminating the interaction between orthographic neighborhood density and word frequency effects in a visual LDT. The effects of density as a function of frequency are now represented differentially in the shift and skew of the underlying RT distributions.

## Author contributions

The author confirms being the sole contributor of this work and approved it for publication.

### Conflict of interest statement

The author declares that the research was conducted in the absence of any commercial or financial relationships that could be construed as a potential conflict of interest.

## Footnotes

^1^Here, the tradeoff between the mu and tau parameters accounts for why the mean interaction effect was very small (see Table 2).

^2^Five parts were plotted in consistency with our recent studies (e.g., Goh et al., 2009; Yap and Seow, 2014) for the ease of comparison across them.

^3^Although activation models, such as McClell and Rumelhart's (1981), can accommodate the present data, one must recall that whether the net effect of neighborhood size is facilitatory or inhibitory depends, within this framework, on the relative values of the parameters governing letter-word excitation, word-word inhibition, and the base activation level associated with word frequency. In a sense, rather than regarding the present data as supporting the model *per se*, it might be more appropriate to regard the data as providing evidence that constrains the future specification of activation models.
