# Supplementary material for: The Influence of Orthographic Neighborhood Density and Word Frequency on Visual Word Recognition: Insights from RT Distributional Analyses
Source: Front Psychol. 2016 Mar 31;7:401. doi: 10.3389/fpsyg.2016.00401 (PMC4814588; doi:10.3389/fpsyg.2016.00401)
Supplement: Supplementary file 1 [file Table1.pdf]

SUPPLEMENTARY MATERIAL

Words			
Low-frequency, low-density	*Low-frequency, high-density*	*High-frequency, low-density*	*High-frequency, high-density*
BLED	BEET	CLUB	BAND
BLOB	BUNK	DISK	BOOK
BROW	CLAP	DOWN	CALL
CHEF	CORK	DROP	CARD
CLOG	DIKE	EACH	CASH
CRIB	DINE	EDGE	CODE
CUFF	GASH	ELSE	COLD
DRIP	GULL	FACT	FILE
DUCT	HUNK	FILM	FIND
FERN	LASH	FISH	FIRE
FUZZ	LENT	FREE	FULL
GLEE	LICE	GIRL	GAME
GNAW	LOOT	HUGE	HEAD
GOWN	MEND	JOIN	HILL
GRUB	MINK	KEPT	HOPE
GULP	MITE	KNEW	KILL
HURL	PAIL	KNOW	LAST
ITCH	PANT	LEFT	LINK
JINX	PECK	MEET	LOVE
JOLT	PEST	MUCH	MARK
KNIT	PORE	NEXT	MIND
LOAF	RAKE	ONCE	MINT
MOTH	RIPE	PLAN	MUST
PIMP	SANK	PLUS	NEAR
PLUM	SEAM	RISK	PART
PUFF	SLAB	RULE	PASS
SMOG	SOCK	STEP	PAST
SMUG	SPAT	STOP	PICK
SNOB	SWAM	SUCH	RACE
SNUG	TAME	TEXT	REAL
STUB	TART	THEM	SAME
SULK	TILE	THIS	SAVE
THAW	VEST	THUS	SENT
THUG	VINE	TOWN	SLOW
TROT	WAIL	TURN	TAKE
TURF	WELT	VOTE	TELL
WEPT	WILT	WALK	TEST
WHIZ		WHEN	WALL
WISP		WITH	WELL
YOKE		YEAH	WIDE

Non-words			
ATCH	GASE	MOME	THOP
ATTS	GEAT	NARL	TOLS
AZED	GERB	NEAF	TORD
BAMS	GIRM	NENS	TOUS
BAPE	GOGS	NERB	TOVE
BARL	GORN	NOID	TRAL
BESS	GOVE	NUGS	TUMB
BIRM	GRIS	NURD	TUST
BISS	GROL	ONGS	VANG
BLEB	GUGS	ORMS	VARC
BLEN	HAMP	PESH	VARP
BLET	HASE	PHOC	VATE
BLEV	HASS	PHYS	VAVE
BLOF	HELT	PIDS	VEAP
BOPE	HEME	POWD	VERM
BRAM	HIGS	PRIF	VIRD
BRIB	HING	PRIL	VONE
BUIP	ISPS	RALD	VUID
CANG	JADS	RASS	VUNE
CANX	JAKE	RERV	WADD
CASS	JARE	RIDS	WAPS
CENS	JEUD	ROSS	WERF
CLUG	JICK	RUDS	WINT
CUSH	JIFT	SADS	WOON
DEND	JUCT	SCUP	WROG
DOMS	JURS	SELT	WROS
DOOT	KAGS	SERB	YAIL
DUMS	KEAR	SIMP	YEVS
DUSH	KIEL	SLAN	YOAT
DUTS	KILE	SMIE	YOPE
EAKS	LAGE	SOVE	YUCH
FARC	LELL	STEA	ZAIR
FEMS	LONS	SULL	ZEAP
FIME	LOOL	SWEP	ZINN
FIPS	LUNK	TAMS	ZOLK
FOMS	MANT	TASS	ZORK
FOWS	MARM	TAZZ	ZOWN
FRAB	MEBS	TEIN	
FUIN	MIDE	THAB	
GABS	MOGS	THOB	
